# Pharmacokinetics and preliminary data on safety of meloxicam subcutaneous extended-release formulation in Sprague–Dawley rats

**DOI:** 10.3389/fvets.2026.1686805

**Published:** 2026-02-20

**Authors:** Avantika Gupta, Harshita Akkaraju, Vignesh Vasudevan, Sruthi Turaga, Lisa C. Rohan, Sravan Kumar Patel, Barry Levinson, Steven Leary, Susan Witham, Imam H. Shaik, Raman Venkataramanan

**Affiliations:** 1 Department of Pharmaceutical Sciences, University of Pittsburgh School of Pharmacy, Pittsburgh, PA, United States; 2 Magee-Womens Research Institute, Pittsburgh, PA, United States; 3 Fidelis Animal Health, North Brunswick, NJ, United States; 4 Department of Pharmacy & Therapeutics, University of Pittsburgh School of Pharmacy, Pittsburgh, PA, United States; 5 Department of Pathology, University of Pittsburgh School of Medicine, Pittsburgh, PA, United States

**Keywords:** exposure, meloxicam suspension, nonsteroidal anti-inflammatory drugs (NSAIDs), safety, subcutaneous-extended release (SC-XR)

## Abstract

**Introduction:**

Nonsteroidal anti-inflammatory drugs (NSAIDs) play a critical role in managing pain and inflammation in veterinary medicine. Meloxicam, a widely used NSAID, is commonly administered as oral or injectable solution but there is a demand for formulations that can be administered less frequently. Our objective was to develop a safe extended-release subcutaneous meloxicam formulation that can be administered less frequently yet maintaining the plasma levels sufficient for adequate response.

**Methods:**

Pharmacokinetics of meloxicam of a subcutaneous suspension [extended release (SC-XR)] formulation was evaluated in Sprague Dawley rats in comparison to intravenous (IV), subcutaneous (SC)] and sustained release (SC-SR), formulations. Dose-escalation and multiple-dosing studies were conducted to assess the drug exposure and safety of the SC-XR suspension formulation.

**Results:**

Pharmacokinetic analysis revealed that the XR suspension formulation achieved prolonged drug release and sustained meloxicam concentrations above 1 µg/ml in plasma for up to 72 hours. Multiple dosing of SC-XR formulation (5mg/mL) at escalating doses of 4 mg/kg, 12 mg/kg, and 20 mg/kg demonstrated minimal drug accumulation. No dose-dependent adverse findings observed in the histological, hematological and organ function marker (AST, ALT, Scr, BUN) assessments.

**Discussion:**

The new meloxicam XR suspension (4mg/kg) was safe and well tolerated. The meloxicam XR suspension represents a potential option for enhancing pain management and compliance in animal care, offering the prospect of less frequent administration for veterinary purposes.

## 1 Introduction

Pain management is critical both in laboratory rats and mice undergoing painful research procedures and in domestic animals requiring treatment. The two primary classes of systemic analgesics used are nonsteroidal anti-inflammatory drugs (NSAIDs) and opioids. Due to variations in therapeutic response and potential side effects, NSAIDs are increasingly used in combination with opioids in multimodal analgesia protocols established for humans (1) and may also be helpful in animals. This approach enhances pain relief while reducing the required doses of individual drugs, thereby mitigating the adverse effects of each drug (2). Although NSAIDs and opioids are both used for postoperative analgesia in rodents, opioid use can present practical challenges, including restricted availability and strict regulatory requirements (3–5). Buprenorphine is the most frequently administered opioid in rodents due to its high analgesic efficacy and comparatively long dosing interval (6, 7). However, NSAIDs are not subject to these regulatory barriers and remain effective in managing mild-to-moderate postoperative pain. In 2000, meloxicam, a benzothiazine NSAID was approved by the U.S. Food and Drug Administration (FDA) for human use, reported anti-inflammatory, antipyretic, and analgesic properties. By selectively inhibiting cyclooxygenase-2 (COX-2), meloxicam reduces inflammation and poses a lower risk of gastric ulceration than non-selective NSAIDs (8–10). With a half-life of nearly 20 h in mice, rats, and sheep, meloxicam is indicated for the treatment of both acute and chronic conditions such as rheumatoid arthritis and ankylosing spondylitis (10, 11). Early rat studies have indicated that meloxicam, when used as the sole analgesic, may adequately alleviate postoperative pain (12). For human use, meloxicam is marketed as Mobic® or generics. Oral suspensions, such as Metacam® and Meloxidyl®, are also available for veterinary use. A compounded sustained-release meloxicam formulation is available for veterinary use (13). A long-acting drug is a medication whose therapeutic effect persists for an extended period due to its inherent pharmacokinetic properties, such as slow absorption, delayed metabolism, and elimination, resulting in sustained activity after administration (14, 15). An extended-release (ER) drug is a pharmaceutical formulation designed to gradually release the active ingredient over a prolonged period, thereby maintaining therapeutic plasma concentrations and reducing dosing frequency through controlled release mechanisms (16).

Conventional pain relief in animals, such as the use of oral medications and repeated injections, presents significant challenges. Frequent dosing can induce stress in rodents, particularly when they resist medication or become anxious after repeated handling. Additionally, inconsistent drug administration may lead to fluctuating plasma drug concentrations, resulting in periods of inadequate pain relief (11). There are currently no FDA-approved long-acting injectable meloxicam formulations available on the market. A long-acting analgesic can address these challenges by providing sustained drug release that maintains therapeutic plasma concentrations for longer durations. Slow-releasing and long-acting analgesics are particularly beneficial for postsurgical recovery, where continuous pain relief supports healing and mobility. It also provides an effective solution for managing chronic pain conditions, such as osteoarthritis, without the need for daily dosing. Given the growing concerns regarding opioid use in veterinary medicine, the development of a long-acting meloxicam formulation represents a significant advancement, offering a safer, more effective, and stress-free alternative to conventional analgesics (11). The goal of this study is to assess if sustained plasma exposure can be achieved following administration of an extended-release (XR) suspension of meloxicam and if dose-dependent adverse effects are observed in histological, hematological, and organ function marker assessments in male Sprague–Dawley rats.

## 2 Materials and methods

### 2.1 Chemicals and reagents

Meloxicam standards were purchased from Toronto Research Chemicals (Toronto, Ontario, Canada). Piroxicam standards were purchased from MilliporeSigma (Burlington, MA, USA). HPLC-grade acetonitrile was purchased from Fisher Scientific (Fair Lawn, NJ, USA). Monobasic potassium phosphate was purchased from Sigma-Aldrich (St. Louis, MO, USA). Ketoprofen (100 mg/mL) was purchased from Zoetis (Parsippany, NJ, USA); Lidocaine HCl (2%) from Hospira Inc. (Lake Forest, IL, USA); Bupivacaine HCl (0.5%) from Fresenius Kabi (Lake Zurich, IL, USA); and Heparin Sodium (1,000 IU/mL) from Sagent Pharmaceuticals (Schaumburg, IL, USA). Blank rat plasma was procured from Equitech-Bio, Inc. (Kerrville, TX, USA). Meloxicam solution was obtained from Covetrus (Portland, ME, USA). The Meloxicam-XR suspension formulation and matching placebo were formulated in-house at the School of Pharmacy, University of Pittsburgh (Pittsburgh, PA, USA). A polymer-based sustained-release (SC-SR) meloxicam formulation was obtained from Wedgewood Connect (San Jose, CA, USA). All other chemicals of the highest purity (analytical grade) were obtained from commercial sources.

### 2.2 Rats

A total of 46 (2-month-old) male Sprague–Dawley (SD) rats (*Rattus norvegicus*), strain Crl: SD, weighing between 250 and 300 grams, were sourced from Charles River Laboratories, Inc. (Wilmington, MA, USA). The rats were housed in an animal facility at the University of Pittsburgh, maintained under a 12-h light/dark cycle, and had unrestricted access to food and water *ad libitum*. The rats were acclimatized to the facility for a minimum of 3 days before the experiment began. All procedures were performed in compliance with the protocol approved by the University of Pittsburgh Institutional Animal Care and Use Committee (IACUC, Protocol #23022712) and adhered to the *Guide for the Care and Use of Laboratory Animals* (17, 18). Daily monitoring included the assessment of physical activity, injection site reactions, and body weight. Animal weight was recorded before administering each dose and once daily thereafter. Physical activity was assessed by routine visual observation of general behavior, posture, mobility, and responsiveness. Injection site reactions were monitored through visual inspection without quantitative scoring, specifically observing signs such as erythema, swelling, or excessive scratching at the injection site.

### 2.3 Study design

This study consisted of two sets of experiments: Experiment 1: a single dose and Experiment 2: multiple doses. For both experiments, subcutaneous dosing (solution, sustained release (SC-SR), and extended release (SC-XR) formulations) was accomplished using a 1 mL syringe and a 23G needle; intravenous (IV) administration of solution was performed using a 1 mL syringe and a 30G needle. The formulations under study, along with a placebo, were administered subcutaneously over the shoulder flanks into the loose skin over the neck. To prevent backflow, light pressure was applied to the injection site for approximately 5 seconds immediately after administration, and no leakage of dosing solution was observed.

#### 2.3.1 Single dose study

The study involved 31 two-month-old male SD rats that were randomly assigned to seven groups. To minimize bias and potential day-related effects, dosing was staggered across days such that animals from different treatment groups were dosed on each study day rather than dosing all animals from the same group on a single day. The sample size of at least four rats per group allowed the estimation of key pharmacokinetic parameters and assessment of inter-animal variability while enabling the identification of consistent exposure trends. Each group received different formulations of meloxicam, including solution [intravenous (IV), subcutaneous (SC)], sustained-release (SC-SR), and extended-release (SC-XR) formulations. IV meloxicam solution was administered via the penile vein. Subcutaneous dosing of all formulations was administered over the shoulder flanks into the loose skin over the neck. SC-XR meloxicam formulations were administered subcutaneously at doses of 2 mg/kg (2 mg/mL) and 4 mg/kg (5 mg/mL). Injection volumes were adjusted according to body weight for each formulation. On average, for a rat weighing approximately 0.26 kg, the administered volume for IV solution, SC solution, SC-SR, and SC-XR (2 mg/mL at a dose of 2 mg/kg and 5 mg/mL formulations) was approximately 250 μL. For SC-XR (2 mg/mL at a dose of 4 mg/kg), an average volume of approximately 500 μL was administered. For the SC-XR formulation at 4 mg/kg with a concentration of 5 mg/mL, including the sterilized formulation, the injection volume was approximately 200 μL.

##### 2.3.1.1 Surgery

Jugular vein cannulation was performed on the first day of the study. Anesthesia was induced with 5% isoflurane (Isoflurane, USP; liquid for inhalation; Piramal Critical Care) and maintained with 2%–2.5% isoflurane and 100% oxygen with a delivery rate of 1 L/min using a nose cone. The depth of anesthesia was assessed using a toe-pinch or corneal reflex, along with continuous monitoring of the respiratory rate. An ophthalmic lubricant (Puralube® vet ointment; Dechra) was applied to the eyes to prevent corneal dryness, and ketoprofen (2.5 mg/kg) was given before surgery as preemptive analgesia. Hair at the surgery site was trimmed using an electric trimmer, and the skin was prepared by alternating povidone-iodine scrub and sterile saline three times, followed by the application of povidone-iodine solution (BETADINE surgical scrub, Povidone-iodine, 7.5%; Purdue Frederick Co.). Once the skin was adequately dry, a small incision was made to expose the jugular vein, allowing precise catheter placement (silicone-tubing-tipped polyethylene 50 tubing; Instech Laboratories, PA, USA). The muscle was irrigated with a local anesthetic solution containing a 50:50 mixture of lidocaine and bupivacaine. The catheter was externalized behind the ear via a small incision in the skin and closed with a plug. Surgery was conducted under aseptic conditions in accordance with the IACUC *Guidelines for Rodent Survival Surgery* (18). The animals regained consciousness 3–5 min after isoflurane was stopped. The study team continuously monitored the rats until they regained their righting reflexes. The animals were kept under light during surgery, and body temperature was maintained. The rats were administered saline to compensate for blood loss.

#### 2.3.2 Multiple dose study

The study involved 16 two-month-old male SD rats that were randomly assigned to four groups. Rats received placebo or 5 mg/mL of the new meloxicam formulation (SC-XR) at escalating doses of 4 mg/kg, 12 mg/kg, and 20 mg/kg. Rats in the 4 mg/kg group received approximately 0.25 mL per injection, those in the 12 mg/kg group received about 0.7 mL, and those in the 20 mg/kg group received around 1 mL of the suspension. Injection volumes were body weight-adjusted, and the placebo group received 1 mL of placebo. The multiple-dose study lasted for 14 days, with doses at 0, 72, and 168 h. In the placebo group, volume of the placebo formulation equivalent to that in the 20 mg/kg group was administered at intervals matching the dosing schedule.

### 2.4 Blood sampling and euthanasia

Blood samples were collected at predetermined time points to evaluate the pharmacokinetics of meloxicam in the single-dose study. For the intravenous (IV) and subcutaneous (SC) dosing groups, blood was drawn at 0, 0.25, 0.5, 1, 2, 4, 8, 12, 24, 48, 72, and 96 h post-administration. For the extended-release (XR) and sustained-release (SR) formulations, blood samples were collected at 0, 1, 2, 4, 8, 12, 24, 48, 72, and 96 h post-dosing. In instances of catheter failure 48 h after implantation, these animals were considered dropouts for the 72-h time point, but were otherwise included for the 96-h time point. At each time point, approximately 200 μL of blood was collected in heparinized tubes (Heparin sodium injection, USP; 1,000 units per ml; Sagent™, Sagent Pharmaceuticals, IL, USA) and immediately centrifuged to separate plasma, which was then transferred to fresh microcentrifuge tubes and stored at −80 °C until analysis by HPLC. For the dose-escalation and multiple-dosing groups, blood samples were collected at 72 and 168 h from the saphenous vein to monitor meloxicam trough concentrations, with a final collection at 336 h prior to euthanasia. Upon study completion, rats were anesthetized under 5% isoflurane for induction, and anesthesia was maintained with 1%–2% isoflurane. Blood was collected via the abdominal aorta, and tissue samples, including liver, small intestines, and skin, were collected for analysis and to ensure the death of the animals. Sections of the skin, liver, and gastrointestinal (GI) tract were fixed in 10% neutral buffered formalin (Richard-Allan Scientific LLC, Epredia Netherlands B.V.) for histopathological evaluation.

The personnel responsible for dosing and sample collection were aware of the group assignments due to the nature of the formulations administered. Outcome assessments, including pharmacokinetic sample analysis and laboratory measurements, were conducted without knowledge of treatment allocation. Data processing and statistical analyses were performed using coded datasets, with group identities revealed only after the completion of the analyses.

### 2.5 Quantitation of meloxicam in rat plasma

#### 2.5.1 Preparation of standards and samples

##### 2.5.1.1 Standard preparation

Meloxicam stock solutions were prepared by accurately weighing and dissolving meloxicam in dimethyl sulfoxide (DMSO) to obtain a primary stock solution of 10 mg/mL. The primary stock solution was serially diluted using a mobile phase [67:33% v/v mixture of potassium phosphate buffer (50 mM, pH 2.15) and acetonitrile] as diluent to generate neat standard solutions with concentrations ranging from 0.1 μg/mL (LLOQ) to 20 μg/mL (ULOQ). Matrix-matched calibration standards and quality control (QC) samples at 0.2, 0.8, 8, and 16 μg/mL were prepared by spiking blank rat plasma with the appropriate concentrations of meloxicam. Piroxicam was used as an internal standard to enhance analytical accuracy and precision. Piroxicam (450 μL) in acetonitrile (1 μg/mL) was added to 50 μL of the standard solution to facilitate protein denaturation and precipitation. The final concentration of piroxicam achieved in all the standard solutions was 0.9 μg/mL.

##### 2.5.1.2 Sample preparation

Samples with higher drug concentrations at specific time points were diluted 5–10 times using blank rat plasma to ensure that the concentrations fell within the standard curve range. Piroxicam (450 μL) in acetonitrile (1 μg/mL) was added to 50 μL of the plasma sample to facilitate protein denaturation and precipitation. The mixture was vortexed thoroughly and centrifuged at 15,000 g for 10 min at 4 °C. Following centrifugation, the clear supernatant was carefully transferred into a clean total recovery vial, and 50 μL was injected into the HPLC column for chromatographic separation and analysis of meloxicam concentrations.

#### 2.5.2 Chromatographic conditions

The mobile phase consisted of a 67:33% (v/v) mixture of potassium phosphate buffer (50 mM, pH 2.15) and acetonitrile. Chromatography was performed using a Waters Alliance e2695 separation module equipped with a Waters Nova-Pak® C_18_ column (5 μm, 150 × 3.9 mm ID) for analyte separation. The column was maintained at 35 °C, and separation was achieved under isocratic elution conditions at a flow rate of 1 mL/min. The injection volume for both standards and samples was 50 μL, with a total run time of 10 min. Meloxicam was detected at 360 nm using a single-wavelength UV detector. Meloxicam and piroxicam eluted with retention times of 7.34 and 4.5 min, respectively, without any interference. The lower limit of quantification (LLOQ) was defined as the analyte concentration producing a response at least 10 times greater than that of the blank sample.

#### 2.5.3 Analytical method validation

The analytical method was validated following the U.S. FDA’s *Bioanalytical Method Validation Guidance for Industry, 2018* (19, 30, 31). The optimized reverse-phase high-performance liquid chromatography (RP-HPLC) method was validated to assess specificity, linearity, sensitivity, precision, accuracy, and robustness. Stability testing included three freeze–thaw cycles (12 h each), storage at 4 °C (refrigerated for 24 h), and room temperature (bench-top stability for 4 h). Performance quality control (QC) samples were compared with freshly prepared calibration curves and QC samples. These studies confirmed the reliability of the analytical method and that meloxicam remained stable under different storage and handling conditions.

##### 2.5.3.1 Accuracy and precision

Accuracy, defined as the closeness of agreement between the reference or theoretical concentration (true value) and the measured concentration (observed value), was evaluated by spiking meloxicam at seven different concentrations (0.1, 0.25, 0.5, 1, 5, 10, and 20 μg/mL). Samples at each concentration were prepared in triplicate to ensure the reproducibility and reliability of the method. Precision, defined as the degree of agreement (or variability) among multiple measurements taken from the same sample under consistent conditions, was evaluated at two levels: repeatability (intra-day precision) and intermediate precision (inter-day precision). Intra- and inter-day precisions were determined by calculating the coefficient of variation across three replicates of quality control (QC) samples across the standard curve, including concentrations at 0.2, 0.8, 8, and 16 μg/mL. Intra-day precision was assessed within the same day, whereas inter-day precision was measured over three consecutive days.

##### 2.5.3.2 Linearity

Linearity was assessed using seven calibration standard concentrations ranging from 0.1 to 20 μg/mL, each prepared in triplicate. Calibration curves were constructed (1/X weighting) by plotting the average peak area ratio of meloxicam over the peak area of piroxicam (internal standard) against nominal meloxicam concentrations (μg/mL). The coefficient of determination (*R*^2^) was calculated to evaluate the method’s linearity and its ability to produce proportional responses across the tested concentration range.

##### 2.5.3.3 Stability

QC samples at two concentration levels (0.8 and 16 μg/mL) were prepared in triplicate and subjected to three stability conditions: refrigerated storage at 4 °C for 24 h, room temperature (RT) for 4 h, and three freeze–thaw cycles, 12 h each cycle. The freeze–thaw stability test involved freezing the samples at 0 °C, thawing them for an hour, and refreezing them for three consecutive cycles to assess any potential degradation due to repeated temperature fluctuations. These stability tests ensured the reliability and consistency of the analytical method over time.

### 2.6 Pharmacokinetic studies

The pharmacokinetic parameters were calculated with a model-independent approach: non-compartmental analysis (NCA) using Phoenix WinNonlin 8.5.2 (Certara, Princeton, NJ, USA). Maximum plasma concentration (C_max_), time to maximum concentration (T_max_), area under the curve to the last quantifiable time point (AUC_₀–t_), area under the curve extrapolated to infinity (AUC_0–inf_), terminal phase elimination rate constant (k), elimination half-life (t_1/2_) volume of distribution during the terminal phase (V) or apparent volume of distribution V/F), and clearance (CL) or apparent clearance (CL/F), were obtained for different treatment groups.

### 2.7 Histopathology

Histopathological evaluations were conducted on liver, skin, and gastrointestinal (GI) tissues collected in dose-escalation study groups (four rats per group) to assess any potential changes in the tissue structure. Tissue samples were fixed in 10% neutral-buffered formalin, paraffin-embedded, and sectioned at 10 μm using a microtome. Tissue sections were stained with hematoxylin and eosin (H&E), and histopathological analysis was performed by microscopic examination. Histological studies were conducted by a veterinary pathologist who was blinded to the study groups. Tissue sections were examined for morphological changes, and a standardized scoring system was used to assess the extent of histopathological alterations in the skin and liver tissues. Scoring was performed based on predefined criteria to quantify tissue damage, inflammation, and other relevant histological features. Overall inflammation and extramedullary hematopoiesis (EMH) were evaluated using a semi-quantitative scoring system ranging from 0 to 4. A score of 0 indicated no inflammation or EMH, 1 indicated minimal findings, 2 indicated mild findings, 3 indicated moderate findings, and 4 indicated severe inflammation or EMH.

### 2.8 Hematology and organ function markers

Hematological and biochemical markers were assessed in rats from the dose-escalation study, including those receiving placebo, 4 mg/kg, 12 mg/kg, and 20 mg/kg of meloxicam SC-XR, to evaluate potential systemic effects. Liver function was assessed by measuring alanine aminotransferase (ALT) and aspartate aminotransferase (AST) levels, whereas kidney function was evaluated using serum creatinine (Scr) and blood urea nitrogen (BUN). Whole blood samples from the terminal bleed were collected for hematology and organ function marker assessment. An automated hematology analyzer (DxH 3; Beckman Coulter, CA, USA) was used to measure white blood cells (WBC), red blood cells (RBC), hemoglobin (HGB), hematocrit (HCT), platelets (PLT), and differential counts. Blood smears were prepared automatically using the DxH Slidemaker Stainer (Beckman Coulter, CA, USA), which aspirates a whole-blood sample, creates a uniform smear on a microscope slide, and stains it using fixatives, buffers, and other solutions according to the manufacturer’s protocol. Organ function markers were analyzed in rat serum using an automated clinical chemistry analyzer (FUJI DRI-CHEM 4000i; Fujifilm Corporation, Tokyo, Japan) with manufacturer-provided reagents following standard operating procedures.

### 2.9 Statistical analysis

Descriptive statistics such as the mean, standard deviation (SD), and coefficient of variation (CV%) were calculated for all pharmacokinetic (PK) parameters to summarize the data distribution. Student *t*-tests were used to assess statistically significant differences between groups. One-way ANOVA tests, along with Brown-Forsythe tests and Bartlett’s tests to test for homogeneity of variance, were performed to compare organ function markers across meloxicam dose groups in dose escalation studies. A *p*-value of <0.05 was considered statistically significant. All statistical analyses were performed using GraphPad Prism Ver. 10.4.1 (GraphPad, MA, USA).

## 3 Results

### 3.1 Safety

The animals used in this study were in good health, exhibiting normal physiological parameters and no signs of distress. They were monitored for any signs of distress or pain [e.g., inactivity, labored breathing, sunken eyes, hunched posture, piloerection/matted fur, abnormal vocalization (when handled), or >20% of baseline body weight loss compared to age-matched controls]. Once-a-day monitoring of body weight indicated a normal growth rate throughout the study, ensuring that the experimental conditions did not induce any physiological stress. As depicted in Table 1, mean rat body weights on day 15 of the study in placebo versus treatment groups of Meloxicam-SC-XR 4 mg/kg, 12 mg/kg, and 20 mg/kg were 336 ± 29 g, 369 ± 12 g, 365 ± 32 g, and 337 ± 15 g, respectively.

**Table 1:** Multiple dosing in rats: body weight and meloxicam trough concentrations.

Day	Weight in grams				Meloxicam Trough Concentration (μg/mL)		
	Placebo (*n* = 4)	4 mg/kg (*n* = 4)	12 mg/kg (*n* = 4)	20 mg/kg (*n* = 4)	4 mg/kg (*n* = 4)	12 mg/kg (*n* = 4)	20 mg/kg (*n* = 4)
1	261 ± 3	268 ± 9	265 ± 11	260 ± 14	—	—	—
4	305 ± 11	309 ± 6	297 ± 20	288 ± 14	3.54 ± 1.1	6.45 ± 2.7	7.36 ± 4.3
8	343 ± 17	346 ± 8	341 ± 27	317 ± 11	1.75 ± 0.7	4.45 ± 2.8	4.06 ± 2.6
15	336 ± 29	369 ± 12	365 ± 32	337 ± 15	0.24 ± 0.1	0.68 ± 1.1	0.82 ± 0.6

Values indicate the mean ± SD.

### 3.2 HPLC assay

The analytical method demonstrated good precision, accuracy, and linearity, confirming its reliability for quantitative analysis. In accordance with the FDA guidance for bioanalytical method development and validation, the performance of calibrators and QCS fell within ± 15% of their nominal concentrations, except for the LLOQ calibrator, which permitted a deviation of up to ± 20% in each validation run. Intra-day precision results show low variability, with a coefficient of variation (CV) ranging from 4.2% at the lowest concentration to 0.9% at the highest, indicating repeatability, as shown in Table 2. Stability studies revealed that the analyte remained stable after three freeze–thaw cycles, 12 h each (0.8 μg/mL and 16 μg/mL, % CV ≤ 2.0 and 0.5, respectively), at 4 °C for 24 h (0.8 μg/mL and 16 μg/mL, % CV ≤ 0.4 and 0.5, respectively), and at room temperature for 4 h (0.8 μg/mL and 16 μg/mL, % CV ≤ 0.2 and 9.3, respectively). The standard curve was linear over the tested range, with an *R*^2^ of 0.9988 (1/X weighted regression). Overall, the method was robust, precise, accurate, linear, and stable for analytical applications.

**Table 2:** Intra-day and inter-day accuracy and precision.

Theoretical conc. (μg/ml)	Calculated conc. (μg/ml)		Accuracy (% recovery)		Precision (% CV)	
	Intra-day	Inter-day	Intra-day	Inter-day	Intra-day	Inter-day
0.2	0.20 ± 0.01	0.20 ± 0.00	100	95	4.20	0.5
0.8	0.85 ± 0.02	0.82 ± 0.04	106	103	2.29	5.1
8	7.69 ± 0.22	8.17 ± 0.42	96	111	2.89	5.2
16	16.45 ± 0.15	16.79 ± 0.65	103	105	0.90	3.9

Mean ± SD values for calculated concentration (μg/ml), accuracy reported as percent recovery, and precision reported as percent coefficient of variation (% CV).

### 3.3 Pharmacokinetic profiles

#### 3.3.1 Single-dose study

**Figure 1:**
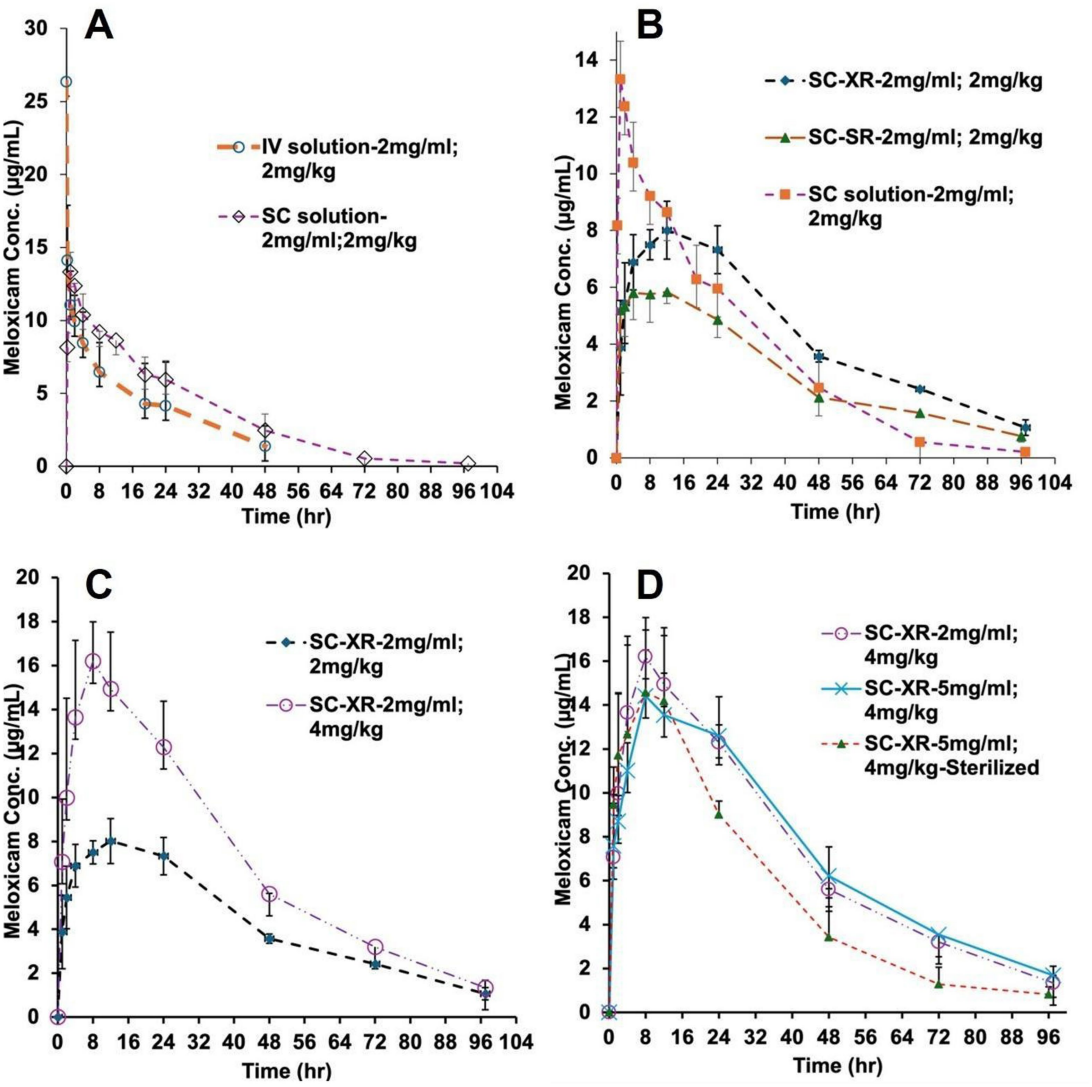
Plasma concentration-time profile of meloxicam in male SD rats after **(A)** IV (*n* = 4) and SC administration (*n* = 4) of meloxicam solution (2 mg/kg); **(B)** SC administration of solution, SR (*n* = 3) and XR (*n* = 4) formulations (2 mg/kg); **(C)** SC administration of meloxicam 2 mg/mL XR formulation dosed 2 mg/kg (*n* = 4) and 4 mg/kg (*n* = 3); and **(D)** SC administration of XR formulations: meloxicam 2 mg/mL (*n* = 7), 5 mg/mL (*n* = 7), and 5 mg/mL, radiation sterilized (*n* = 5) dosed 4 mg/kg. Data represent mean plasma concentrations, and error bars indicate standard deviation.

Figure 1 illustrates the plasma concentration vs. time profiles of various meloxicam formulations. Figure 1A shows the plasma concentrations after IV administration of meloxicam at a 2 mg/kg dose. The maximum concentration (C_max_) of 26.36 ± 4.8 μg/mL was reached immediately after dosing, which was followed by a decline over time with a half-life of 18.4 ± 6.9 h, and the observed AUC was 315 ± 177 μg·h/mL. SC administration of the solution formulation at the same dose showed an absorption phase, indicating an increase in plasma levels over time and reaching a maximum concentration (C_max_) of 13.3 ± 1.3 μg/mL at a time (T_max_) of 1.5 ± 0.7 h. The estimated half-life of meloxicam was 20.9 ± 7.9 h, and the estimated AUC was 385 ± 75 μg·h/mL (Table 1). Meloxicam concentrations declined rapidly during the post-absorption phase after SC administration. Sustained-release (SC-SR) and extended-release (SC-XR) formulations showed a markedly prolonged absorption phase, with T_max_ values of 10 ± 5.5 and 18.7 ± 9.2 h, respectively. These formulations resulted in a significantly lower C_max_ (6.8 ± 0.8 and 7.9 ± 0.4 μg/mL) compared to the solution. Figure 1B demonstrates that the SC-XR and SC-SR formulations resulted in lower C_max_ and sustained plasma concentrations over a prolonged time compared to the SC solution. Even though a higher C_max_ was attained upon SC administration of the solution, meloxicam trough levels were considerably lower than those of SC-SR and SC-XR formulations. SC-XR formulation showed a significantly (Two-sample *t*-tests, *p* = 0.0006) higher AUC (480.6 ± 64.2 μg·h/mL) among SC groups, suggesting enhanced overall exposure.

Figure 1C and Table 1 show that dose escalation of the SC-XR formulation from 2 to 4 mg/kg resulted in a dose-proportional increase in plasma concentration and prolonged systemic exposure with an increase in AUC from 480.6 ± 64.2 to 800.3 ± 155.9 μg·h/mL, and the concentration at 48 h (C_48_) also increased proportionally. Dose-normalized pharmacokinetic parameters of meloxicam from the SC-XR formulation showed consistent AUC_₀–t_, AUC_₀–∞_, and C_max_ values after 2 mg/kg and 4 mg/kg doses, indicating linear pharmacokinetics. Figure 1D demonstrates that increasing the meloxicam concentration in the XR formulation from 2 mg/mL to 5 mg/mL, while maintaining the same dose, did not result in altered pharmacokinetic parameters. No statistically significant differences were observed in the pharmacokinetic parameters between the sterilized and unsterilized Meloxicam-XR formulations.

#### 3.3.2 Multiple-dose study

The pharmacokinetic parameters observed after multiple doses of the Meloxicam-XR formulation demonstrated dose-dependent systemic exposure with minimal accumulation over time. Trough plasma concentrations on day 3 were dose-dependent, with higher doses (12 mg/kg and 20 mg/kg) maintaining elevated levels compared to 4 mg/kg; concentrations on day 3 were 3.54 ± 1.1 μg/mL (4 mg/kg), 6.45 ± 2.7 μg/mL (12 mg/kg), and 7.36 ± 4.3 μg/mL (20 mg/kg) (Table 3). Compared to the single-dose study, where concentration at 72 h was ~3.5 μg/mL (4 mg/kg SC-XR), the multiple-dose study also resulted in a C_72_ of ~3.54 μg/mL (4 mg/kg), indicating similar exposure and pharmacokinetics in rats. The plasma exposure data suggest that meloxicam follows predictable linear pharmacokinetics, with higher doses maintaining proportionally greater systemic exposure.

**Table 3:** Pharmacokinetic parameters of the investigated meloxicam formulations in male SD rats.

PK Parameters	IV Solution (2 mg/ml)	SC Solution (2 mg/ml)	SC-SR Polymer in Solution (2 mg/mL)	SC-XR Suspension (2 mg/mL)	SC-XR Suspension (2 mg/mL)	SC-XR Suspension (5 mg/ml)	SC-XR Suspension (5 mg/ml) Sterilized
*N*	4	4	3	4	3	7	5
Dose (mg/kg)	2	2	2	2	4	4	4
Half-life (h)	18.4 ± 6.9	20.9 ± 7.9	25.2 ± 1	26.4 ± 3.4	23.2 ± 4.3	19.6 ± 4.8	18.4 ± 7.9
AUC_0-t_ (μg.hr/mL)	274.1 ± 151.3	320.3 ± 9.8	309.9 ± 30.8	439 ± 61.1	754.1 ± 161.2	607.4 ± 218.2	532.2 ± 60.2
AUC_0-inf_ (μg.hr/mL)	315 ± 177.1	385 ± 75.2	337.7 ± 38.7	480.6 ± 64.2	800.3 ± 155.9	644.4 ± 250.0	550.2 ± 62.9
CL or CL/F (mL/h/kg)	8.3 ± 4.6	5.3 ± 1.0	6.0 ± 0.7	4.2 ± 0.5	5.1 ± 0.9	7 ± 2.7	7.3 ± 0.8
Vd (mL/kg)	186.5 ± 51.1	154.1 ± 28.8	216.5 ± 20.5	160 ± 24.3	172.3 ± 50.1	190.3 ± 62.6	195.5 ± 89.3
C_max_ (μg/mL)	26.4 ± 4.8	13.3 ± 1.3	6.8 ± 0.8	7.9 ± 0.4	16.2 ± 1.8	15.9 ± 2.5	15.1 ± 2.5
T_max_ (h)	0	1.5 ± 0.7	10 ± 5.5	18.7 ± 9.2	8 ± 0.0	9.3 ± 2.3	10.4 ± 2.2
C_48_ (μg/mL)	1.4 ± 1.4	2.5 ± 0.6	2.1 ± 0.0	3.6 ± 0.2	5.6 ± 0.02	6.2 ± 1.3	3.4 ± 1.4
C_72_ (μg/ml)	2.00 ± 0.2	0.6 ± 0.0	1.6 ± 0	2.4 ± 0.0	3.2 ± 0.7	3.5 ± 0.5	1.3 ± 0.7
C_96_ (μg/mL)	0.8 ± 1.1	0.2 ± 0	0.8 ± 0.2	1.1 ± 0.3	1.3 ± 0.3	0.6 ± 0.3	0.8 ± 0.4

Values indicate the mean ± SD.

**Figure 2:**
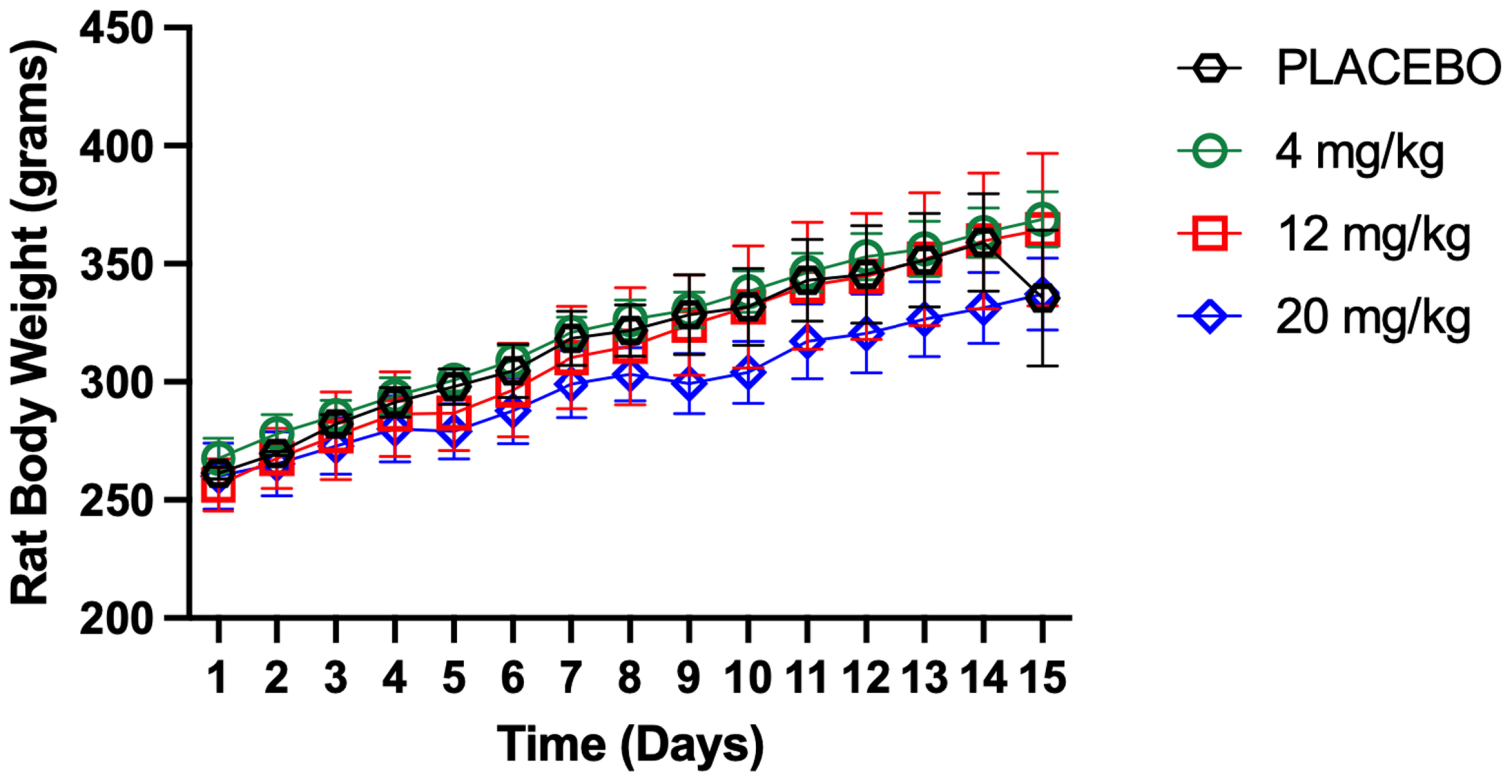
Body weight measurements (g) over time for placebo-treated rats and those receiving meloxicam at 4, 12, or 20 mg/kg (each dose group, *n* = 4) of SC-XR suspension. Data represent the mean rat body weight, and error bars indicate the standard deviation.

As shown in Figure 2 and Table 3, no significant differences in body weight were observed between placebo-treated rats and those receiving meloxicam at 4, 12, or 20 mg/kg. Multiple paired *t*-tests were performed, and none of the comparisons reached statistical significance (*p* > 0.05). Rat body weight gain remained consistent in both placebo and SC-XR groups, which was similar to the weight gain reported in normal healthy SD rats (10 weeks, 380 ± 35 g) (20), suggesting that multiple doses of Meloxicam-XR administration did not impact the overall health of animals.

### 3.4 Histopathology

**Figure 3:**
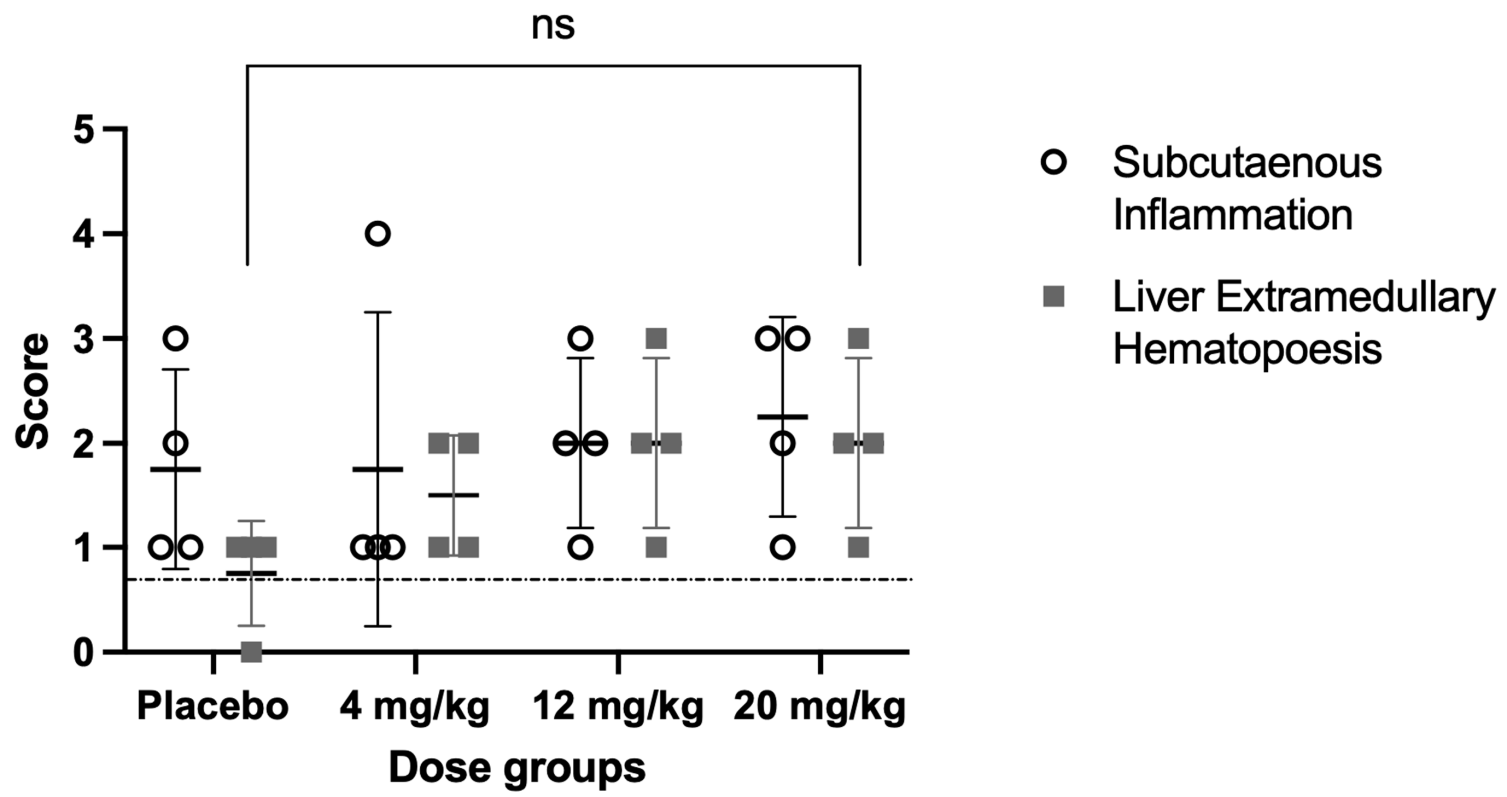
Scores for the degree of subcutaneous inflammation and liver extramedullary hematopoiesis (EMH) across different dose groups. Data are presented as the mean ± SD. A score of 0 indicated no inflammation or no EMH; 1 indicated minimal findings; 2 indicated mild findings; 3 indicated moderate findings; and 4 indicated severe inflammation or severe EMH. The broken line depicts the reference SQ inflammation score (historical control) in healthy male SD rats (29). Statistical analysis was performed using one-way ANOVA (subcutaneous inflammation: *p* = 0.899; liver EMH: *p* = 0.078). Homogeneity of variances was assessed using Bartlett’s test.

The histopathological evaluation revealed that subcutaneous inflammation, characterized by mixed inflammatory cell infiltrates as well as fibroblast proliferation and edema, was comparable across all groups, including both placebo and Meloxicam-SC-XR-treated animals, as depicted in Figure 3. Mean ± SD values of subcutaneous inflammation scores for placebo and treatment groups 4 mg/kg, 12 mg/kg, and 20 mg/kg were 1.75 ± 0.9, 1.75 ± 1.5, 2.00 ± 0.8, and 2.25 ± 0.9, respectively. There was no significant difference in subcutaneous inflammation between the groups (One-way ANOVA, *p* = 0.899). This indicated that the meloxicam formulation did not elicit an injection site reaction, supporting its safety for subcutaneous administration. In contrast, a mild increase in liver extramedullary hematopoiesis (EMH), which featured white or blood cell precursors, was present in the portal areas or sinusoids in meloxicam-treated groups, particularly at higher doses (12 mg/kg and 20 mg/kg), compared to the placebo group. Mean ± SD values of EMH scores for placebo and treatment groups 4 mg/kg, 12 mg/kg, and 20 mg/kg were 0.75 ± 0.5, 1.5 ± 0.6, 2.00 ± 0.8, and 2.00 ± 0.8, respectively. There was no significant difference in EMH between the groups (One-way ANOVA, *p* = 0.078). While this may indicate a potential systemic hematopoietic effect, the changes remained mild and were not indicative of severe pathology.

### 3.5 Hematology and organ function markers

The evaluation of liver and kidney function markers confirmed that meloxicam administration did not induce hepatic or renal toxicity across all tested doses. As shown in Table 4, the measured AST and ALT levels were not elevated beyond normal physiological values in rats and therefore do not suggest hepatic injury or impaired liver function, even at higher doses (12 mg/kg and 20 mg/kg). A one-way ANOVA was employed to compare AST levels across the placebo and different dose groups. The analysis showed no significant differences among the group means [F (3,12) = 0.465, *p* = 0.712]. Similarly, for ALT, one-way ANOVA showed no significant differences among group means [F (3,12) = 1.098, *p* = 0.388]. Serum creatinine (Scr) and blood urea nitrogen (BUN) levels remained stable, suggesting that renal function was unaffected by repeated doses of Meloxicam-XR. A one-way ANOVA was used to compare BUN levels and Scr across placebo and different dose groups. The analysis showed no significant differences among the group means, F (3,12) = 1.852, *p* = 0.192, and F (3,12) = 3.259, *p* = 0.059, respectively. Hematological assessments revealed no significant alterations in hematocrit (HCT) levels, confirming that Meloxicam-XR did not adversely affect red blood cell parameters (One-way ANOVA, *p* = 0.158). While white blood cell (WBC) counts were slightly lower in the 4 mg/kg and 12 mg/kg groups, the highest dose (20 mg/kg) showed a slight increase, potentially reflecting individual variability rather than a consistent treatment effect. Differences in WBC counts across dose groups were not significant (One-way ANOVA, *p* = 0.085). None of the values for hematological, liver, and kidney function markers were elevated beyond the normal physiological values reported for age-matched male SD rats (21) (see Table 4).

**Table 4:** Liver and kidney damage marker profile and hematology in rats treated with higher doses of Meloxicam-SC-XR suspension, 4 mg/kg.

Marker	Parameter (Normal range) (23)	Placebo (*n* = 4)	4 mg/kg (*n* = 4)	12 mg/kg (*n* = 4)	20 mg/kg (*n* = 4)	*p*-value
Liver and kidney damage markers	ALT (28–40 IU)	38.25 ± 3.3	31.50 ± 4.7	35.75 ± 9.2	32.75 ± 3.3	0.387
	AST (87–114 IU)	51.50 ± 2.4	51.25 ± 5.5	54.25 ± 5.1	51.00 ± 4.1	0.712
	Scr (0.5–0.6 mg/dL)	0.24 ± 0.02	0.24 ± 0.01	0.21 ± 0.01	0.24 ± 0.02	0.059
	BUN (13–16 mg/dL)	16.25 ± 0.5	15.5 ± 0.6	14.5 ± 1.7	15.25 ± 1.0	0.192
Hematology	HCT (41.2–47.3%)	39.87 ± 0.5	40.33 ± 2.8	41.03 ± 1.2	38.25 ± 1.0	0.159
	WBC (10.09–14.01 cells10^3^/μL)	6.85 ± 4.9	4.65 ± 2.0	3.75 ± 1.2	9.68 ± 3.1	0.085

ALT, alanine aminotransferase; AST, aspartate aminotransferase; Scr, serum creatinine; BUN, blood urea nitrogen; HCT, hematocrit; WBC, white blood cells. Values indicate mean ± SD. Statistical analysis: One-way ANOVA.

**Figure 4:**
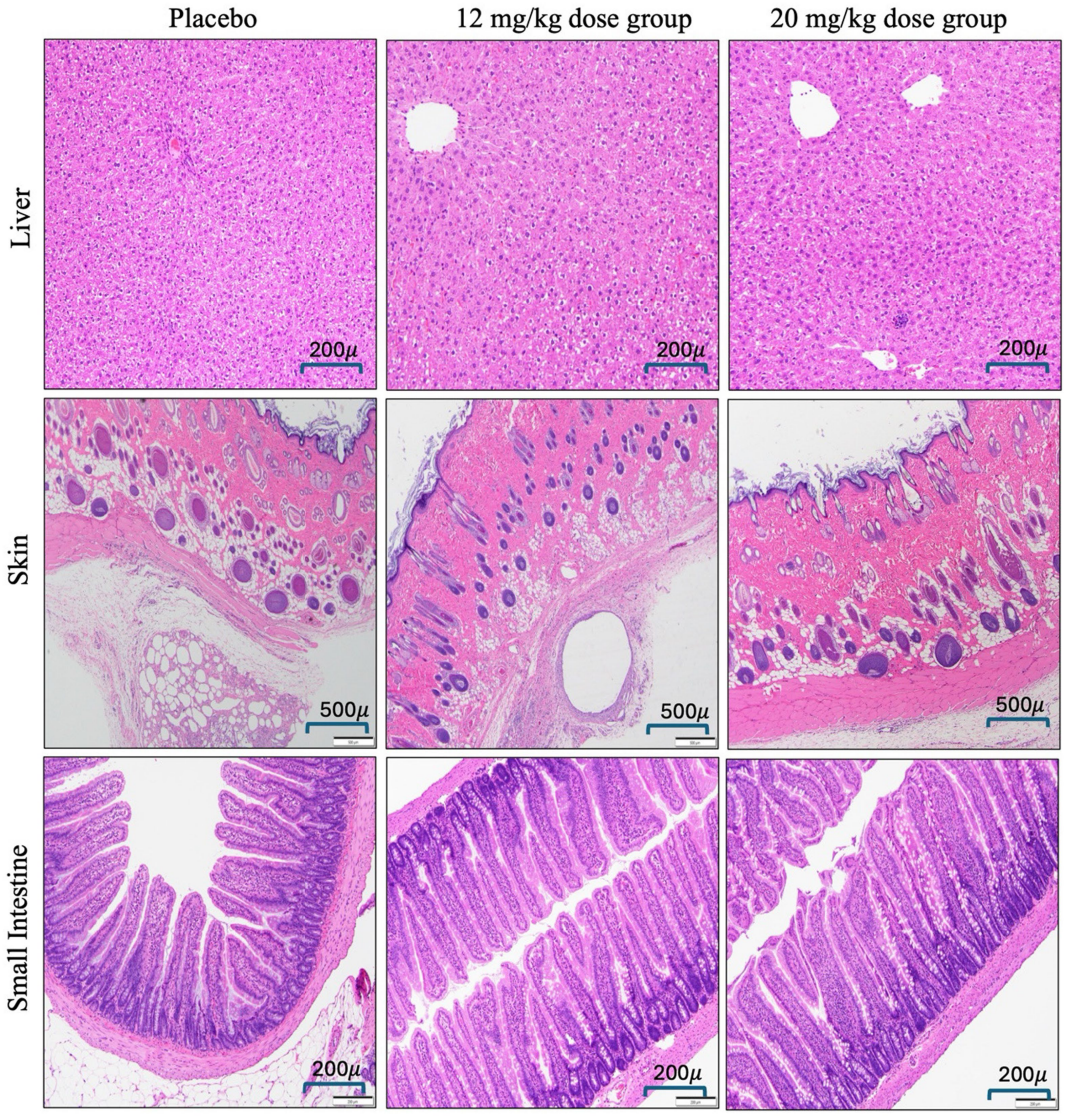
Microscopic images of hematoxylin and eosin-stained tissues across different dose groups of Meloxicam-SC-XR suspension (Liver and small intestine tissues examined under a light microscope at 10X magnification, while skin samples were observed at 4X magnification).

## 4 Discussion

A pharmacy-compounded sustained-release formulation from ZooPharm (now a subsidiary of Wedgewood Pharmacy) is the only available sustained-release formulation of meloxicam (Melox-SR, a polymer-based injectable solution) (13). Although studies investigating Meloxicam-ER at 4 mg/kg in male Sprague–Dawley rats reported injection site reactions (22), which may be likely due to its formulation composition, the manufacturer recommends administering Meloxicam-ER to rats at a dose of 4 mg/kg every 72 h (23) and that further evaluation is needed (11). However, institutional guidelines advise caution with extended-release meloxicam formulations, noting that independent studies in rodents have not demonstrated efficacy beyond 24 h post-administration (24). While the manufacturer of polymer-based SR formulations recommends dosing every 72 h, critical gaps remain regarding dose proportionality and systemic availability. Given this information, our research becomes increasingly relevant in evaluating the pharmacokinetics and safety of a newer extended-release formulation for meloxicam.

This study aims to assess the safety only for the newly developed XR formulation of meloxicam and the pharmacokinetics of Meloxicam-SC-XR suspension, and to characterize its prolonged release, ensuring sustained plasma concentrations while minimizing dosing frequency in SD rats. Modulating the absorption rate from the formulation, such as a sustained-release dosage form, has the potential to achieve longer-lasting therapeutic effects. SC doses of XR (extended release) and SR formulations (2 mg/mL; 2 mg/kg dose) exhibited prolonged plasma concentrations compared to the solution, indicating prolonged absorption from the injection site. Lower C_max_ and higher C_min_ values were observed for both SR and XR formulations compared to the solution. A lower C_max_ is important in clinical settings, as the adverse effects associated with NSAIDs are linked to maximum concentrations. The modified release formulation reduced the propensity for adverse events due to lower plasma levels. Although both SC-XR and SC-SR formulations maintain therapeutic drug levels longer than the solution, indicating their potential for reducing the dosing frequency, the SR formulation shows a quicker decline, presumably suggesting a shorter duration of action compared to the SC-XR formulation. Additionally, SC-XR achieved a higher C_max_ than SC-SR, indicating a higher rate of absorption while maintaining the extended-release characteristics. The SC-XR formulation (2 mg/mL) showed a significantly higher AUC than the SC-SR formulation, suggesting higher systemic exposure. Moreover, a dose-proportional increase in plasma levels as well as exposure was observed with a higher dose (4 mg/kg) when compared to SC-XR (2 mg/kg), indicating dose linearity of the SC-XR formulation. This suggests that increasing the dose increased systemic drug exposure and maintained elevated drug levels for a longer duration. The pharmacokinetic profiles obtained from the 2 mg/mL vs. 5 mg/mL SC-XR formulation show that the profiles of the 4 mg/kg dose for both strengths overlap, indicating that increasing the concentration of meloxicam in the formulation from 2 mg/mL to 5 mg/mL does not alter the pharmacokinetic profile when administered at the same dose. Overall, these results indicate that higher doses provide higher systemic exposure, and the sterilization process does not alter pharmacokinetics, making the radiation-sterilized XR formulation suitable for clinical or veterinary applications. The plasma concentration of meloxicam estimated to be effective for analgesia is approximately 1 μg/mL (11, 25–28). Consistent with this threshold, our data demonstrated that the Meloxicam-XR formulation maintained plasma concentrations above the therapeutic level for up to 72 h. Notably, the half-life (19–23 h) remained relatively consistent, suggesting that prolonged drug exposure was primarily driven by the extended-release characteristics of the formulation, rather than by changes in systemic elimination.

Multiple doses of SC-XR formulation (5 mg/mL) at escalating doses of 4 mg/kg, 12 mg/kg, and 20 mg/kg demonstrated minimal drug accumulation. Meloxicam concentrations in the plasma collected on day 4 (C_72_) showed a clear dose–response relationship, with higher doses (12 mg/kg and 20 mg/kg) maintaining higher levels than 4 mg/kg. However, 4 and 11 days after the second dose (days 7 and 15), the trough levels declined consistently across all doses, suggesting effective drug clearance and limited accumulation. The current study was designed to study 2 doses; if additional doses were to be continued every 72 h, the plasma concentrations at steady state would be above the pain relief threshold. Additionally, the observed concentration differences between the different dosing regimens highlight the potential for further dose optimization to balance therapeutic effectiveness and safety, thereby minimizing the risk of excessive drug accumulation.

This study has several limitations that should be acknowledged. Only male Sprague–Dawley rats were included; therefore, potential sex-related differences in pharmacokinetics and tolerability were not assessed. An untreated control group and a separate control group for repeated blood sampling were not included because the primary objective of the study was pharmacokinetic characterization, for which such controls are not routinely employed. Histopathological evaluation was limited to selected organs, and assessment of additional organs was not performed. Furthermore, systematic clinical assessments of tolerability were not conducted.

The *in vivo* evaluation supported the safety of the XR formulation for meloxicam. The XR formulation did not show any differences in animal body weight gain among the different dose groups compared to the placebo groups. Additionally, the animal growth rate was similar to that of age-matched naïve SD rats (20). Tissue histopathology showed that the Meloxicam-SC-XR formulation is well-tolerated, with no significant inflammatory reactions at the injection site, further reinforcing its safety profile at repeated doses. The hematological analysis indicated the safety of the Meloxicam-XR formulation, ensuring that chronic administration does not cause changes in hematology or organ function. HCT levels were similar across the treatment groups, suggesting no impairment in erythropoiesis or red blood cell turnover, reinforcing long-term tolerability. Additionally, WBC counts stayed within the expected limits and did not surpass the upper threshold of normal (10.09–14.01, cells10^3^/μL) (21), indicating that meloxicam or inactive ingredients in the suspension formulation are safe upon repeated dosing. The levels of liver and kidney function/injury markers, ALT, AST, BUN, and serum creatinine, remained within normal limits, further confirming the safety of the newly developed Meloxicam-XR formulation. Together, these findings support the use of the Meloxicam-XR formulation for sustained analgesic coverage with flexible dose adjustment based on clinical needs.

## 5 Conclusion

This study provides a comprehensive evaluation of subcutaneous (SC) meloxicam formulations, highlighting the pharmacokinetics and safety of both solution and extended-release formulations. The Meloxicam-SC-XR formulation achieves a balance between rapid absorption and prolonged activity, making it suitable for sustained delivery of meloxicam. Meloxicam-SC-XR may be preferred for long-term treatment, reducing the need for frequent dosing while maintaining plasma levels above the pain relief threshold. With polymer-based sustained-release (SC-SR), despite offering a long duration of action, drug exposure may be variable owing to differences between compounded scripts. These findings suggest that the SC-XR (5 mg/mL; 4 mg/kg dose) formulation is optimal for long-term drug release, reducing dosing frequency while maintaining estimated effective plasma concentrations (11). Collectively, these findings demonstrate sustained plasma exposure following the administration of an extended-release suspension of meloxicam, with no dose-dependent adverse findings observed in the limited histological, hematological, and organ function marker assessments. Further studies are needed to determine its applicability to larger animals, such as companion animals (dogs and cats), making this study relevant for additional veterinary applications.

## Data Availability

The original contributions presented in the study are included in the article, further inquiries can be directed to the corresponding author.
